# Effects of replacing soybean meal with cotton protein and benzoic acid supplementation on intestinal morphology, ileal microbiota, and metabolomics in young pigeons

**DOI:** 10.3389/fmicb.2026.1783579

**Published:** 2026-07-31

**Authors:** Qingqing Lu, Yingying Yao, Haiying Li, Lihuan Deng, Run Wu

**Affiliations:** College of Animal Science, Xinjiang Agricultural University, Urumqi, China

**Keywords:** benzoic acid, cotton protein, intestinal enzyme activity, intestinal flora, intestinal tissue morphology, metabolome

## Abstract

This study investigated the effects of substituting soybean meal with cotton protein and supplementing with benzoic acid on the intestinal health of young pigeons. A total of 240 healthy young pigeons aged 28 days were randomly divided into three groups: the basal diet group (Group A), the diet group substituting 5% soybean meal with cotton protein (Group B), and the diet group supplemented with 105 mg/kg benzoic acid in Group B (Group C). The formal experiment lasted 42 days. The results showed that in terms of intestinal enzyme activity, compared with Group A, the amylase activity and trypsin activity in Groups B and C were significantly increased (*P* < 0.05), with higher amylase activity in Group B and higher trypsin activity in Group C. In terms of intestinal development, the ileal length and pH values of each intestinal segment in Group A were significantly higher than those in the treatment groups (*P* < 0.05). In terms of intestinal tissue morphology, compared with Group A, the villus height of the duodenum and jejunum in Groups B and C was significantly increased (*P* < 0.05), whereas the villus height of the ileum and crypt depth were significantly decreased (*P* < 0.05). Compared with Groups A and C, the crypt depth of the jejunum in Group B was significantly increased (*P* < 0.01). There were no significant differences in the α-diversity of the ileal microbiota among the groups, but the composition changed: compared with Group A, the abundance of the Firmicutes phylum in Group C was significantly increased (*P* < 0.05), while the Lactobacillus genus was significantly decreased in Groups B and C (*P* < 0.05). Metabolomics identified 66 differential metabolites, involving multiple metabolic pathways such as amino acid synthesis and ABC transporters. In summary, under the experimental conditions, substituting soybean meal with cotton protein and supplementing with benzoic acid can improve digestive enzyme activity, intestinal morphology, and the abundance of the Firmicutes phylum, thereby accelerating amino acid synthesis metabolism and promoting the intestinal health of pigeons.

## 1 Introduction

China has long relied on imported soybean meal as the main source of protein. The fluctuation of soybean price and supply directly affects the feed cost and the development of animal husbandry. In order to reduce the dependence on soybean meal, researchers have developed a variety of plant protein substitutes, such as cottonseed meal, rapeseed meal and peanut meal. Among them, cotton meal is a potential alternative protein source due to its high nutritional value (He et al., 2025; Nafady et al., 2025). As the world’s major cotton producing country, China produces a large number of high-quality cotton every year, and also produces a rich by-product-cottonseed. Cottonseed is not only an important source of oil, but also contains a rich source of plant protein, namely cottonseed protein. Cotton meal is rich in protein (40–50%), lysine, methionine and other amino acids required by humans and animals, and has antioxidant and immunomodulatory functions (Zhao et al., 2025). However, cottonseed protein contains a substance called free gossypol, which is toxic to monogastric animals (such as poultry) and is called antinutritional factor (Yan et al., 2025). After long-term accumulation of free gossypol in the body, it significantly affects the structure and balance of the ileum flora of the pigeons, and the change of this flora is directly related to the digestion and absorption of the pigeons and intestinal health (Xie et al., 2024; Wang et al., 2025a).

Benzoic acid is an aromatic acid, which not only has broad-spectrum anti-bacterial properties, but also can be used as an acidifier and preservative. When benzoic acid is used as an acidifier, it can neutralize the environment caused by alkaline substances (Khukhodziinai et al., 2024). When benzoic acid is used as a preservative, it can reduce the production of bacteria and thus play a protective role (Zhang Q. et al., 2022). Moreover, benzoic acid can promote the degradation of free gossypol in animals, and the mechanism mainly involves two pathways. One is the direct binding pathway. Benzoic acid binds to plasma proteins, which in turn affects the protein binding state of free gossypol and promotes its metabolic clearance. The second is the indirect intervention pathway. Benzoic acid binds to liver detoxification enzymes and inhibits the key step of the conversion of free gossypol to gossypol glucuronic acid. By inhibiting this main detoxification pathway, the concentration of free gossypol is increased in reverse, but combined with the scavenging effect of the previous pathway, the total free gossypol level is finally reduced (Wang et al., 2025b). However, there is still a lack of research on the effect of benzoic acid supplementation on the intestinal flora of squabs, and the specific effect of benzoic acid supple-mentation on the intestinal health of squabs is not yet clear.

Currently, studies on the effects of cotton meal substitutes for soybean meal and benzoic acid on poultry have primarily focused on broilers and layer chickens, with limited reports on their combined application. Consequently, The purpose of this experiment was to reveal the feasibility of replacing soybean meal (42% crude protein content) with cotton meal (43.5% crude protein content) and supplementing benzoic acid in the diet of young pigeons by analyzing the intestinal enzyme activity, intestinal length and intestinal pH value, intestinal tissue morphology, ileal flora and differential metabolites of young pigeons, and to increase the abundance of Firmicutes in the combination of the two, regulate intestinal microecology, replace growth-promoting antibiotics, and improve overall health.

## 2 Materials and methods

### 2.1 Experimental design

A total of 240 28-day-old healthy white king pigeons with similar body weight were selected and reared under the same conditions. They were randomly divided into 3 groups with 8 replicates in each group and 10 squabs in each replicate. The grouping design is as follows: Group A was fed with corn-soybean meal basal diet; group B used 5% cotton meal to replace the same amount of soybean meal; on the basis of group B, group C was added with 105 mg/kg benzoic acid. The experiment included a 7-day pre-feeding period and a 42-day formal period. The basic feed was formulated according to the nutritional requirements of juvenile pigeons, with its composition and nutritional levels shown in Table 1.

**TABLE 1:** Composition and nutritional level of basic diet for young pigeons (Wind-dried Basis).

Staple	Content		
	Group A	Group B	Group C
Raw grain composition			
Corn	25.00	25.00	25.00
Wheat	5.00	5.00	5.00
Pea	25.00	25.00	25.00
Sorghum	5.00	5.00	5.00
Granular material			
Corn	19.80	19.80	19.80
Secondary powder	0.80	0.80	0.80
Sodium bicarbonate	0.40	0.40	0.40
Salt	0.40	0.40	0.40
Yeast powder	2.00	2.00	2.00
Soybeanmeal	12.60	11.97	11.97
Cotton protein	0	0.63	0.63
Calcium bicarbonate	0.60	0.60	0.60
Methionine	0.16	0.16	0.16
Lysine	0.24	0.24	0.24
Limestone (0∼2 mm)	0.80	0.80	0.80
Benzoic acid	0	0	0.04
Soybean oil	0.40	0.40	0.40
Premix	1.80	1.80	1.80
Total	100.00	100.00	100.04
Nutrient levels			
ME/ (MJ/kg)	11.65	11.52	11.52
CP (%)	15.72	15.89	15.89
Ca (%)	1.20	0.51	0.51
P (%)	0.42	0.32	0.32
Lys (%)	0.77	0.85	0.85
Met (%)	0.36	0.21	0.21
Trp (%)	0.14	0.16	0.16

The premix provides the following nutrients per kilogram of feed: Biotin 1.2 mg; Folic acid 19 mg; Nicotinamide 690 mg; Pantothenic acid 320 mg; Vitamin A 200,000–330,000 IU; Vitamin D 80,000–165,000 IU; Vitamin E 380 IU; Vitamin B1 65 mg; Vitamin B2 180 mg; Vitamin B6 90 mg; Cu 30–830 mg; Fe 2,600–25,000 mg; Zn 1,500–4,000 mg; Mn 1,700–5,000 mg; I 30–160 mg. The nutritional levels in the basic feed table of White-feathered King Pigeon are all calculated values. The composition of the feeding health sand for White-feathered King Pigeon is: Medium-coarse sand 40.0%; Yellow mud 18.0%; Shell powder 27.5%; Calcined gypsum 7.0%; Charcoal powder 3.5%; Alum 1.5%; Gentian 1.0%; Licorice 1.5%.

### 2.2 Sample collection

#### 2.2.1 Sample preservation

The end of the feeding experiment, after a 12-h fast, two meat pigeons were randomly selected from each replicate of Groups A, B, and C. The duodenal contents were collected post-slaughter for measurement of intestinal digestive enzyme activity. Segments of the intestine (duodenum, jejunum, ileum) were collected for histological sectioning. The ileal contents were rapidly collected and stored in 2 mL sterile cryovials, rapidly frozen in liquid nitrogen, and preserved at −80°C for intestinal microbiota and metabolomics analysis. In this study, 30 samples were collected for metabolomics analysis and 30 samples for intestinal microbiota determination.

### 2.3 Determination of intestinal indexes

#### 2.3.1 Amylase activity

Amylase unit definition: Each milligram of protein in the tissue was incubated with the substrate at 37°C for 30 min, and the hydrolysis of 10 mg of amylase was defined as an amylase activity unit.

#### 2.3.2 Trypsin activity

The absorbance OD value was measured at 253 nm, and the absorbance OD value A1 at 30 s was recorded. The absorbance OD value A2 is recorded after 20 min and 30 s.

As shown in Table 2, all the above indicators were detected by ELISA kit purchased from the Institute of Biotechnology.

**TABLE 2:** Biochemical testing.

Testing company	Item	Sample	Production batch number
Shenggong Bioengineering (Shanghai) Co., Ltd.	β-Amylase Activity Detection Kit	Duodenal contents	D799332-0100
Shenggong Bioengineering (Shanghai) Co., Ltd.	Trypsin activity detection kit	Duodenal contents	D799684-0100

Determination of the length of each intestinal segment: After slaughter, the duodenum, jejunum and ileum tissues were taken for length measurement with a tape measure.

Determination of intestinal pH value: The end of the experiment, the intestinal contents were taken out and the intestinal pH value was determined. The pH values of duodenum, jejunum and ileum contents of white king pigeons were measured by pH meter, and the data of two sites were recorded and the average value was calculated.

### 2.4 Intestinal histomorphology

The prepared intestinal tissue sections were placed under a 10-fold optical microscope, and the villus height and crypt depth were observed, and the ratio of the two (villus height VH/crypt depth CD) was calculated.

Villus height (VH): from the top of the villi to the base of the villi, 3 values were observed in each section and each field of view, and the average value was calculated.

Crypt depth (CD): the distance from the bottom of the intestinal gland to the base opening between the two villi. Each slice was observed for 3 values in each field of view, and the average value was calculated.

### 2.5 16 SrRNA gene amplicon sequencing

After DNA extraction, the 16SrRNA V4 variable region was amplified using the bacterial 16SrRNA universal sequence. Primer sequences were screened, PCR amplification procedures were performed, and libraries were constructed. After the library was qualified, sequencing was used.

### 2.6 Non-target metabolomics and data processing

Based on liquid chromatography-mass spectrometry (LC-MS) technology, n-on-targeted metabolomics research is carried out. The experimental process mainly includes: sample metabolite extraction, LC-MS/MS detection and data analysis. The first three QCs before injection were used to monitor the instrument status before injection and to balance the chromatography-mass spectrometry system. QC was inserted in the middle of sample detection to evaluate the stability of the system during the whole experiment, and data quality control analysis, principal component analysis (PCA), partial least squares discriminant analysis (PLS-DA) were carried out to reveal the differences in metabolic patterns between different groups.

### 2.7 Statistical analysis

The experiment was organized by Excel software, and one-way ANOVA was performed on the digestive enzyme activity and intestinal tissue morphology of pigeons using SPSS 26.0 software. Duncan’s method was used for multiple comparisons between groups. All results are presented as mean ± standard deviation. *P* < 0.05 is considered significant, *P* > 0.05 is considered no significant and *P* < 0.01 is considered extremely significant. Microbial analysis of the ileum was performed according to the QIIME1 workflow, including sequence denoising or OTU clustering. Subsequently, QIIME1 software and custom scripts were utilized to analyze samples at various taxonomic levels (phylum and genus). Univariate analysis of variance was conducted using SPSS 19.0, with multiple comparisons performed via the Duncan method. Results were presented as mean ± standard deviation of mean to summarize overall profiles. Alpha diversity indices for each sample were then evaluated using QIIME1 software, R language, and SPSS 19.0. Intestinal tissue sections were examined using DSAssistant software for image analysis, and measurements were performed using ImageJ software. The metabolome was analyzed using ultra-high performance liquid chromatography (UHPLC) coupled with high-resolution mass spectrometry. Differential metabolites were screened based on the criteria of VIP > 1.0, FC > 1.2 or FC < 0.833, with a *P*-value < 0.05.

## 3 Results

### 3.1 Effects of cotton protein and benzoic acid on intestinal digestive enzyme activities in pigeons

As shown in Table 3, compared with group A, the intestinal amylase activity and trypsin activity in group B and group C were significantly increased (*P* < 0.05).

**TABLE 3:** Effects of casein substituting soybean meal and supplementing benzoic acid on the intestinal digestive enzyme activity of young pigeons (U/g).

Item	Group A	Group B	Group C
Amylase activity	15.39 ± 1.57^c^	23.78 ± 1.84^a^	20.23 ± 0.76^b^
Tryptic activity	1.77 ± 0.26^c^	5.84 ± 0.63^b^	6.43 ± 0.86^a^

Data in the same column without letters or the same letter superscripts mean no significant difference (*P* > 0.05), different letter superscripts mean significant difference (*P* < 0.01 as highly significant, *P* < 0.05 as significant). Values with different lowercase superscript letters indicate a significant difference (*P* < 0.05), and values with different uppercase superscript letters indicate an extremely significant difference (*P* < 0.01).

### 3.2 Effects of cotton protein and benzoic acid on intestinal length and intestinal pH value of pigeons

As shown in Table 4, the ileal length in Group A was significantly increased compared to Groups B and C (*P* < 0.05). No significant differences were observed in the lengths of duodenum and jejunum between groups A, B, and C (*P* > 0.05). In terms of intestinal pH, the duodenal, jejunal, and ileal pH values in Group A were significantly higher than those in Groups B and C (*P* < 0.05).

**TABLE 4:** Effects of cotton protein substituting soybean meal and supplementing benzoic acid on gut length and gut pH of young pigeons.

Item	Group A	Group B	Group C
Duodenal length (cm)	15.9 ± 0.13	12.84 ± 0.11	14.64 ± 0.76
Jejunum length (cm)	46.16 ± 7.46	44.23 ± 8.98	44.47 ± 8.54
Length of ileum (cm)	23.63 ± 4.62^a^	20.41 ± 2.58^b^	20.75 ± 3.86^b^
Duodenal pH	5.63 ± 0.96^a^	4.94 ± 0.25^b^	5 ± 0.01^b^
Jejunal pH	6 ± 1.46^a^	5.13 ± 0.34^b^	5.25 ± 0.68^b^
pH of ileum	7.44 ± 0.81^a^	6.69 ± 0.60^b^	6.13 ± 1.02^b^

Data in the same column without letters or the same letter superscripts mean no significant difference (*P* > 0.05), different letter superscripts mean significant difference (*P* < 0.01 as highly significant, *P* < 0.05 as significant). Values with different lowercase superscript letters indicate a significant difference (*P* < 0.05), and values with different uppercase superscript letters indicate an extremely significant difference (*P* < 0.01).

### 3.3 Effects of cotton protein and benzoic acid on intestinal villus height and crypt depth of pigeons

**FIGURE 1:**
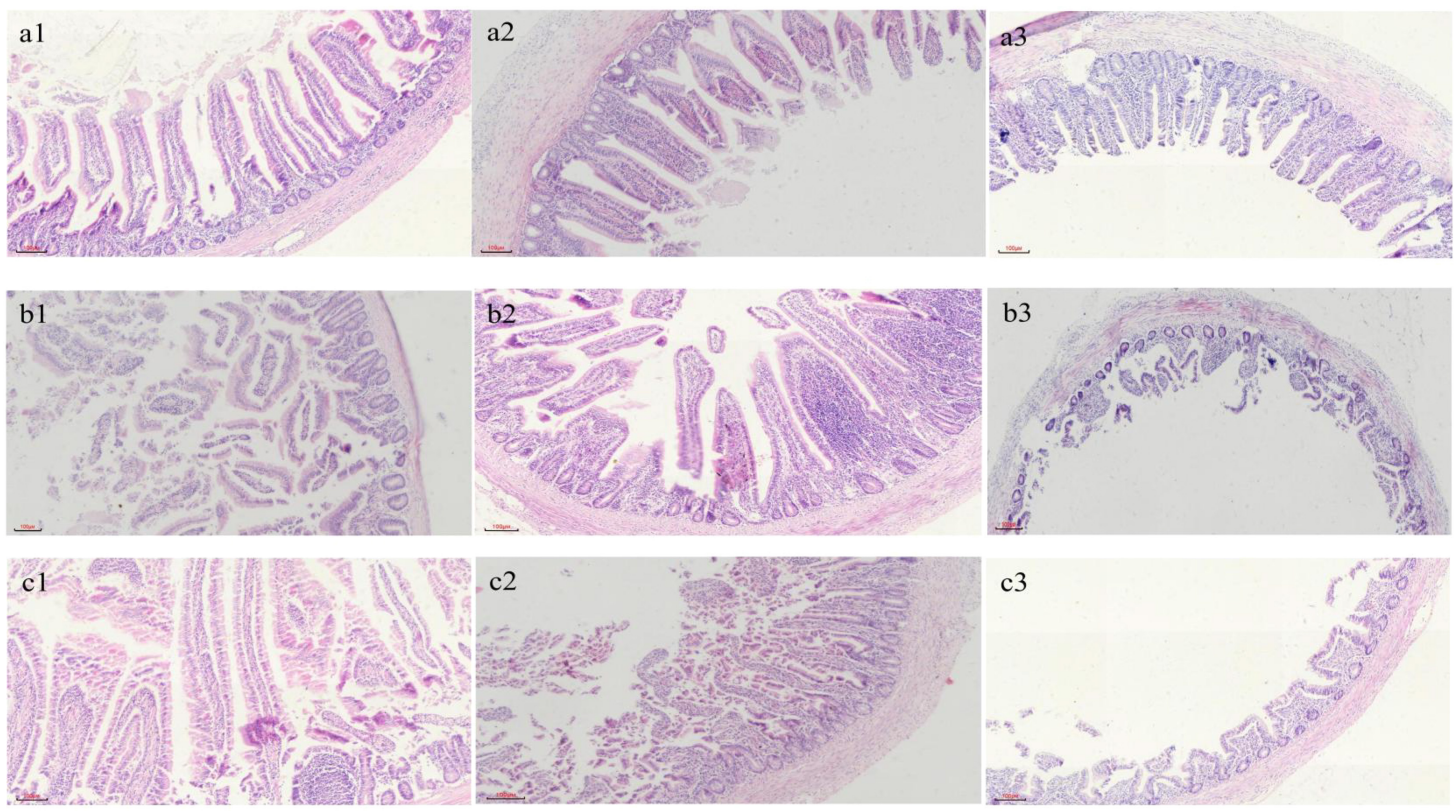
Tissue sections of duodenum, jejunum and ileum of white-feathered king pigeon (HE staining, ×10). **(a1–a3)** Represent the duodenal, jejunum, and ileal tissue sections from the control group; **(b1–b3)** correspond to those from the cotton protein-substituted soybean meal group; **(c1–c3)** are from the benzoic acid supplementation group.

As shown in Figure 1 and Table 5, in the duodenum, compared with Group A, the villus height in Groups B and C was significantly increased (*P* < 0.05), and the villus height in Group C was significantly higher than that in Group B (*P* < 0.05). Compared with Group A, the villus-to-lumen ratio in Group C was significantly increased (*P* < 0.05). In the jejunum, compared with Group A, the villus height in Groups B and C was significantly increased (*P* < 0.05), and the villus height in Group B was significantly higher than that in Group C (*P* < 0.05). Compared with Groups A and C, the crypt depth in Group B was significantly increased (*P* < 0.01). Compared with Group A, the villus-to-lumen ratio in Group B was significantly increased (*P* < 0.05). In the ileum, compared with Group A, the villus height and crypt depth in Groups B and C were significantly decreased (*P* < 0.05).

**TABLE 5:** Effect of casein substituting for soybean meal and supplementing benzoic acid on the morphology of gut tissue in young pigeons (μm).

Item	Group A	Group B	Group C
Duodenum			
Villi height	657.53 ± 21.00^c^	737.05 ± 31.92^b^	833.70 ± 32.22^a^
Depth of crypt	114.06 ± 10.70	116.06 ± 12.76	123.89 ± 8.13
Heterochromatous ratio	5.82 ± 0.64^b^	6.43 ± 0.82^ab^	6.76 ± 0.52^a^
Jejunum			
Villi height	305.40 ± 42.39^c^	437.67 ± 43.30^a^	332.48 ± 26.00^b^
Depth of crypt	64.94 ± 3.00^B^	71.56 ± 6.21^A^	61.68 ± 6.27^B^
Heterochromatous ratio	4.72 ± 0.77^b^	6.18 ± 0.95^a^	5.45 ± 0.74^ab^
Ileum			
Villi height	228.25 ± 12.71^a^	151.46 ± 6.04^b^	158.98 ± 5.82^b^
Depth of crypt	64.81 ± 3.54^a^	45.80 ± 1.09^b^	45.27 ± 4.86^b^
Heterochromatous ratio	3.53 ± 0.31	3.31 ± 0.14	3.54 ± 0.34

Data in the same column without letters or the same letter superscripts mean no significant difference (*P* > 0.05), different letter superscripts mean significant difference (*P* < 0.01 as highly significant, *P* < 0.05 as significant). Values with different lowercase superscript letters indicate a significant difference (*P* < 0.05), and values with different uppercase superscript letters indicate an extremely significant difference (*P* < 0.01).

### 3.4 Effects of cotton protein and benzoic acid on the relative abundance of intestinal flora in pigeons

**FIGURE 2:**
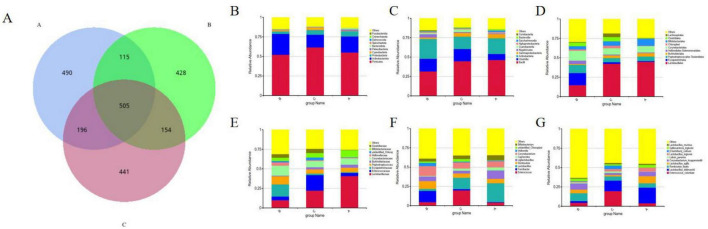
Effects of replacing soybean meal with cotton protein and supplementing benzoic acid on the relative abundance of intestinal flora in pigeons. **(A)** Venn diagram. **(B)** Phylum-level species abundance histogram. **(C)** Class level species abundance histogram. **(D)** Column diagram of species abundance at order level. **(E)** Column diagram of species abundance at family level. **(F)** Column diagram of genus level species abundance. **(G)** Species-level species abundance histogram. A: Basic diet group; B: 5% cotton protein instead of soybean meal group; C: 5% cottonseed protein instead of soybean meal + 105 mg/kg benzoic acid group.

In Figure 2, we show the effect of replacing soybean meal with cotton protein and supplementing benzoic acid on the relative abundance of intestinal flora in pigeons. In the Venn diagram (Figure 2A), the number of OTUs shared by group A, group B and group C was 505, the number of OTUs unique to group A was 490, the number of OTUs unique to group B was 428, and the number of OTUs unique to group C was 441. The number of OTUs shared by group A and group B was 115, the number of OTUs shared by group A and group C was 196, and the number of OTUs shared by group B and group C was 154. The number of OTUs in group B and group C was lower than that in group A, and the number of OTUs in group C was higher, indicating that the supplementation of 105 mg/kg benzoic acid in the pigeon diet had the best effect on the bacteria and quantity of the ileum flora of the pigeons.

At the phylum level, we identified the top 10 different phylums of the pigeon ileum flora in this experiment (Figure 2B). All the content samples in group A, group B and group C showed almost the same community structure, and Firmicutes, Actinobacteria and Proteobacteria were the dominant flora. Compared with group A, the relative abundance of Firmicutes in the ileum flora of group C was significantly increased (*P* < 0.05). Compared with group B, the relative abundance of Actinobacteria in the ileum of pigeons in group A and group C was significantly lower (*P* < 0.05).

At the class level, we identified the first 10 different classes of the ileum flora of young pigeons in this experiment (Figure 2C). The microbial communities in group A, group B and group C were dominated by Bacillus and Clostridium, and the overall structure showed certain diversity and functional diff-erentiation. The relative abundance of Bacillus in group A, B, and C was significantly different (*P* < 0.05).

At the order level, we identified the top 10 different orders of the pigeon ileum flora in this experiment (Figure 2D). The dominant groups of microbial communities in groups A, B and C were concentrated in Lactobacillus. It indicates that the grouping treatment may have a directional regulation effect on the community structure of Lactobacillus and its accompanying groups. Compared with group A, the relative abundance of Lactobacillus in the microbial community supplemented with benzoic acid was significantly increased (*P* < 0.05).

At the family level, we identified the top 10 different families of the ileum flora of young pigeons in this experiment (Figure 2E). The dominant groups of the microbial communities in groups A, B, and C focused on the Lactobacillus family. It indicated that the grouping treatment may have a directional effect on the abundance of Lactobacillaceae and the dominance of the whole community. Compared with group A, the relative abundance of Lactobacillaceae in themicrobial community was significantly decreased (*P* < 0.05), whether cotton protein replaced soybean meal or supplemented with benzoic acid.

At the genus level, in this study, we identified the top 10 different genera in the ileal microbiota of juvenile pigeons (Figure 2F). The dominant microbial communities in Groups A, B, and C were predominantly concentrated in the genus Lactobacillus. The dominant taxa were mainly four categories: Lactobacillus, Enterococcus, Romboutsia, and Bifidobacterium, with Lactobacillus accounting for the highest proportion, followed by decreasing order. Compared to Group A, the relative abundance of Enterococcus in the ileal microbiota of Group C juvenile pigeons was significantly increased (*P* < 0.05). Compared to Group A, the relative abundance of Turicibacter in the ileal microbiota of Group B juvenile pigeons was significantly increased (*P* < 0.05). Compared to Group A, the relative abundance of Lactobacillus in the ileal microbiota of both Group B and Group C juvenile pigeons was significantly decreased (*P* < 0.05).

At the species level, we identified the top 10 different species of the pigeon ileum flora in this experiment (Figure 2G). The distribution of microbial groups in group A, group B and group C was mainly concentrated in lactic acid bacteria, and the overall structure had certain diversity and functionality. Compared with group A, the relative abundance of Enterococcus columbae in the microbial community of cotton protein replacing soybean meal and supplementing benzoic acid was significantly increased (*P* < 0.05). Compared with group A, the relative abundance of Lactobacillus johnsonii in the microbial community of cotton protein replacing soybean meal and supplementing benzoic acid was significantly decreased (*P* < 0.05).

### 3.5 Effects of cotton protein and benzoic acid on alpha diversity of intestinal flora in pigeons

**FIGURE 3:**
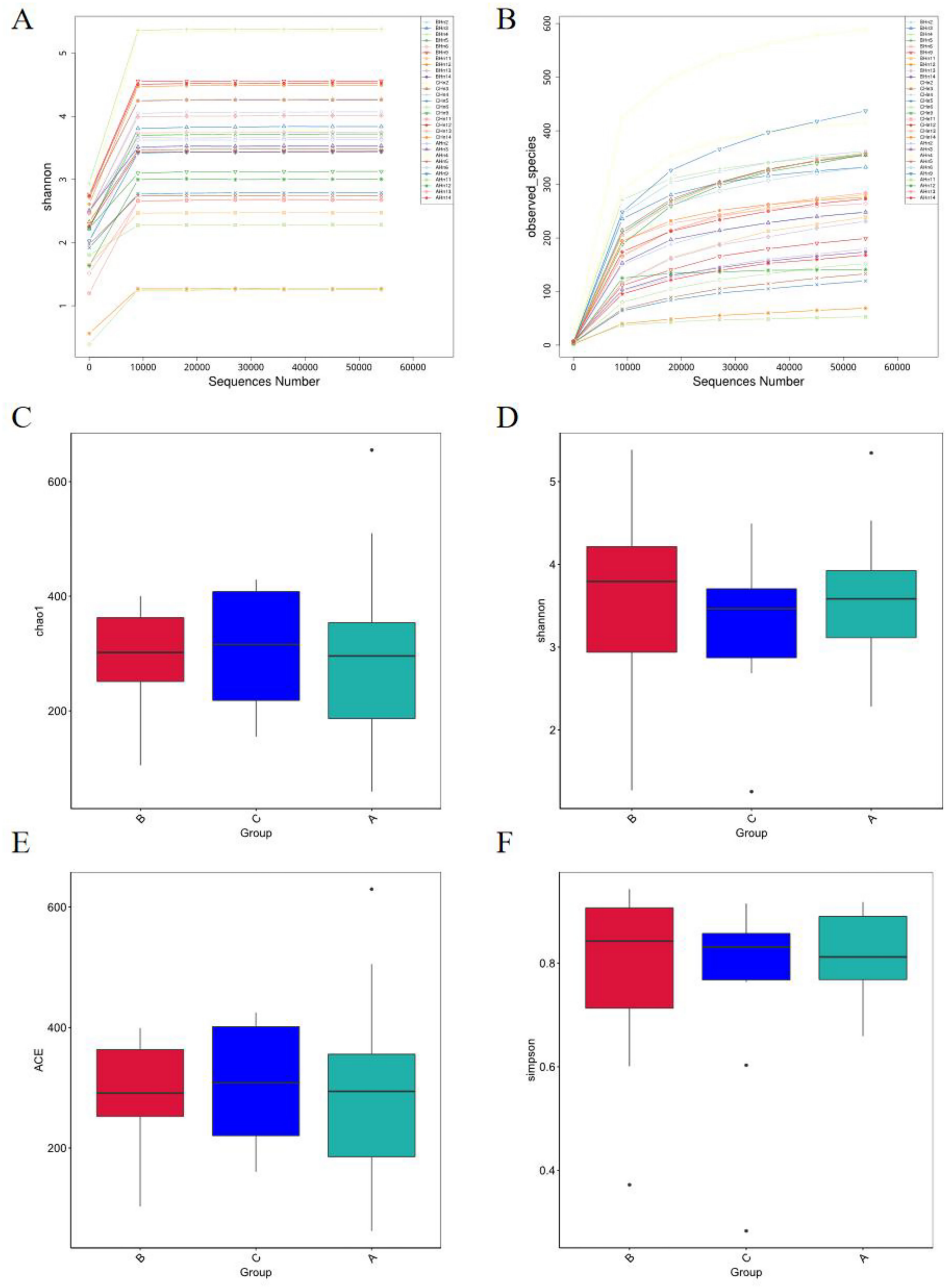
Effect of replacing soybean meal with cotton protein and supplementing benzoic acid on alpha diversity of intestinal flora in pigeons. **(A,B)** α diversity dilution curve. **(C)** α diversity Chao1 index diagram. **(D)** α diversity Shannon index. **(E)** α diversity ACE index. **(F)** α diversity Simpson index. A: Basic diet group; B: 5% cotton protein instead of soybean meal group; C: 5% cottonseed protein instead of soybean meal + 105 mg/kg benzoic acid group.

Figure 3 shows the effect of replacing soybean meal with cotton meal and supplementing benzoic acid on the alpha diversity of intestinal flora in pigeons. We randomly selected a certain amount of sequencing data and counted their α diversity index values. Figures 3A,B show that the dilution curve of each group of samples in this experiment gradually tends to be flat with the increase of sequencing sequences, indicating that more sequencing sequences produce relatively few OTUs, indicating that the sequencing depth is reasonable. When the curve tends to be flat or reaches the platform period, it can also be considered that the sequencing depth has basically covered all the species in the sample, and additional sequencing data cannot find more OTUs. (After the data volume of abscissa sequencing reached 10,000, the dilution curve of each treatment group basically reached a gentle level with the increase of sequencing depth).

We analyzed the difference of α diversity index between groups by Tukey test. In the Chao1 index, group A produced a large outlier. The minimum value of the box plot generated in group B was 157.48, while the minimum values of the box plot generated in group A and group C were 134.28 and 148.22, respectively. Therefore, the box plot of group B is in the highest position. In addition, the quartile range of the boxplot generated by group B was 6.02, while the quartile range of the boxplot generated by group A and group C was 7.02 and 7.32, indicating that the dispersion degree of group B was the smallest and the stability of the results in α diversity was the best (Figure 3C). In the Shannon index, group A produced a larger outlier, and group C produced a smaller outlier. The minimum value of the box plot generated in group B was 1.3, while the minimum values of the box plot generated in group A and group C were 2.3 and 2.8, respectively. Therefore, the box plot of group B is at the lowest position. In addition, the interquartile range of the boxplot generated by group B is 1.09, while the interquartile range of the boxplot generated by group A and group C is 0.77 and 0.79, indicating that group B has the largest dispersion (Figure 3D). In the ACE index, group A produced a large outlier. The minimum value of the box plot generated in group A was 130.54, while the minimum values of the box plot generated in group B and group C were 147.25 and 156.00, respectively. Therefore, the box plot of group A is at the lowest position. In addition, the interquartile range of the boxplot generated by group B is 5.60, while the interquartile range of the boxplot generated by group A and group C is 7.70 and 6.20, indicating that the dispersion of group B is the smallest (Figure 3E). In the Simpson index, Group B generated a single minor outlier, while Group C produced two minor outliers. The box plot of Group B showed a minimum value of 0.67, compared to 0.71 for Group A and 0.76 for Group C, indicating Group B’s lowest position. Additionally, Group C’s box plot had a interquartile range (IQR) of 0.13, whereas Groups A and B exhibited IQRs of 0.15 and 0.16, respectively, demonstrating Group C’s minimal dispersion (Figure 3F).

### 3.6 Effects of cotton protein and benzoic acid on β diversity of intestinal flora in pigeons

**FIGURE 4:**
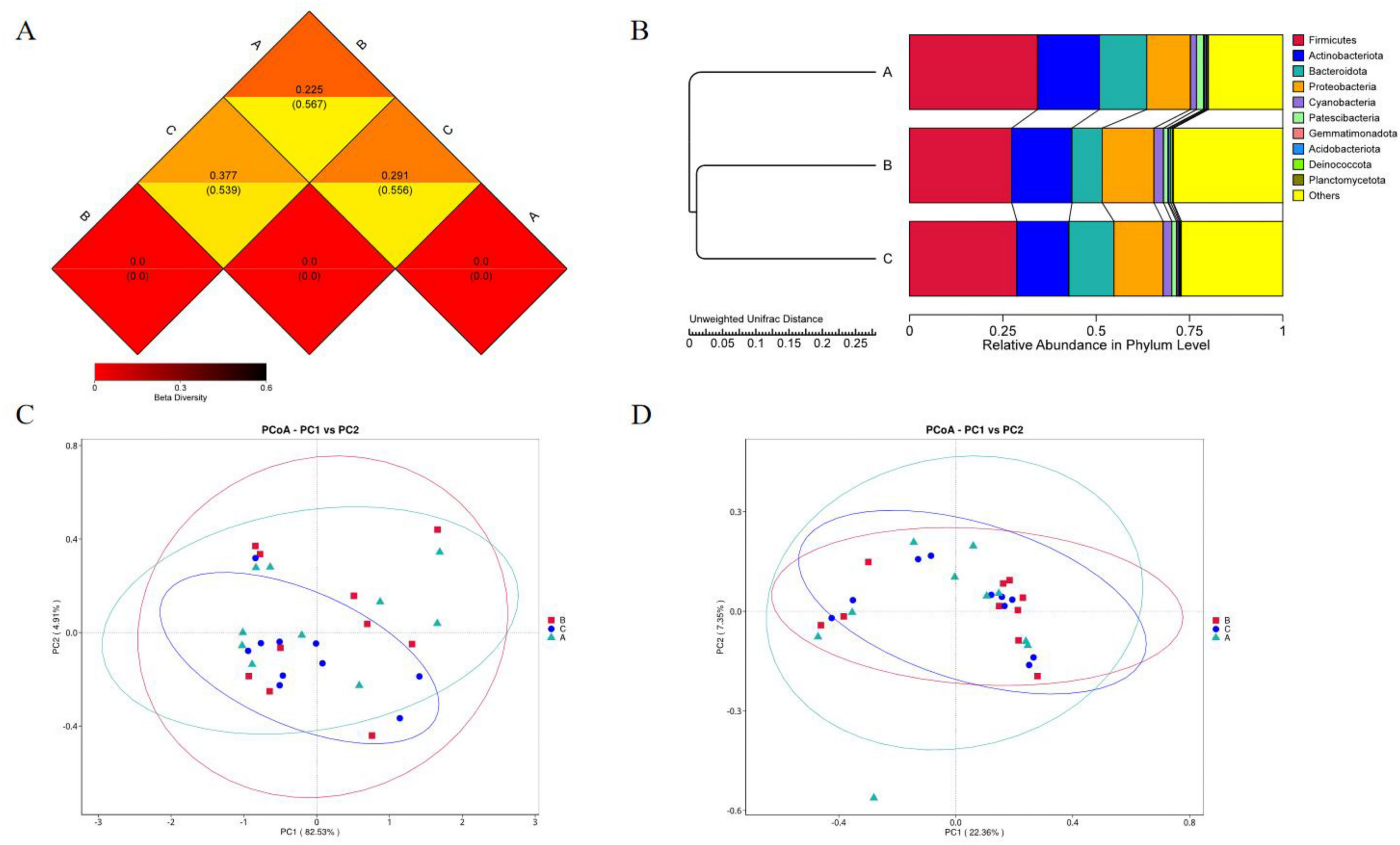
Effect of replacing soybean meal with cotton protein and supplementing benzoic acid on β diversity of intestinal flora in pigeons. **(A)** β diversity index Unweighted unifrac distance matrix heat map. **(B)** UPGMA cluster tree based on Weighted Unifrac distance. **(C,D)** Principal coordinate analysis (PCoA) based on OTUs level. **A:** Basic diet group; **B:** 5% cotton protein instead of soybean meal group; **C:** 5% cottonseed protein instead of soybean meal + 105 mg/kg benzoic acid group.

Figure 4 shows the effect of replacing soybean meal with cotton meal and supplementing benzoic acid on the β diversity of intestinal flora in pigeons. The Beta diversity between group B and group C was 0.377, which was the highest among all groups, indicating that the community composition difference between group B and group C was the largest (Figure 4A). Between group A and group C, Beta diversity was 0.291. Between group A and group B, Beta diversity was 0.225, which was the lowest among the groups, indicating that the difference in community composition between group A and group B was relatively minimal.

In order to study the similarity between different samples, we construct the clustering tree of samples by clustering analysis of samples. The community composition of group A, group B, and group C was dominated by Firmicutes (relative abundance was close to 0.5 and above), followed by Proteobacteria (relative abundance was 0.25–0.3). These two phyla are the core constituent groups of the three communities, and the relative abundance of other phyla (such as Actinobacteria, Cyanobacteria, etc.) is low (Figure 4B).

The results of principal coordinate analysis (PCoA) based on OTUs level are shown in the figure. Principal coordinate 1 is the representative contribution rate of total microorganisms detected, in which the contribution rates of ileum contents were 82.53 and 22.36%, respectively, and the contribution rates of principal coordinate 2 were 4.91 and 7.35%, respectively. Among them, the ileum samples of group A, group B and group C were close, and the community composition was not significantly different (Figures 4C,D).

### 3.7 Effects of cotton protein and benzoic acid on relative abundance cluster analysis of PICRUSt2 functional annotation of intestinal flora in pigeons

**FIGURE 5:**
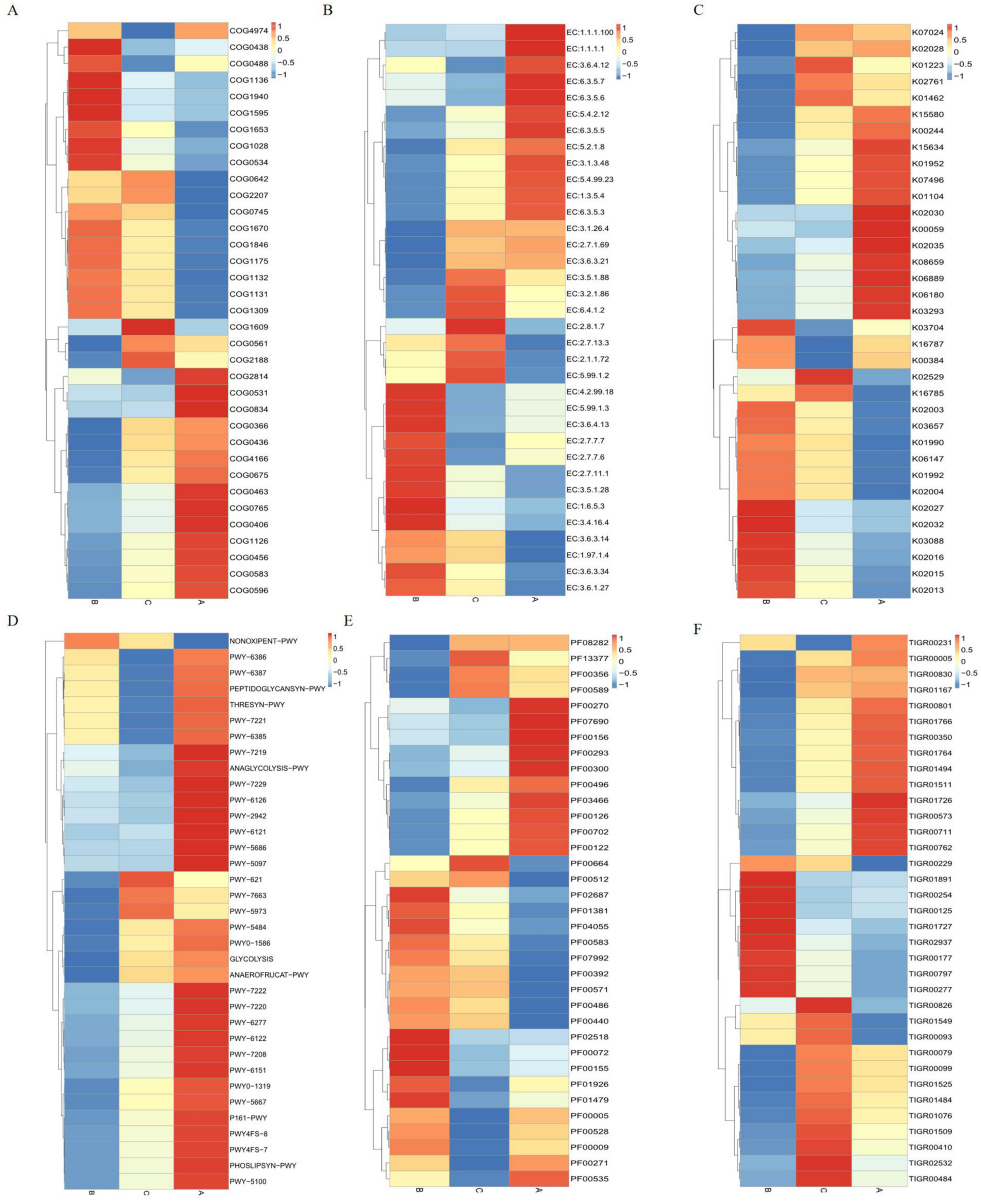
The effect of replacing soybean meal with cotton protein and supplementing benzoic acid on the relative abundance cluster analysis of PICRUSt2 functional annotation of pigeon intestinal flora. **(A)** COG gene function clustering heat map. **(B)** EC gene function clustering heat map. **(C)** KO gene function clustering heat map. **(D)** PWY gene function clustering heat map. **(E)** PF gene function clustering heat map. **(F)** TIGR gene function clustering heat map. A: Basic diet group; B: 5% cotton protein instead of soybean meal group; C: 5% cottonseed protein instead of soybean meal + 105 mg/kg benzoic acid group.

Figure 5 Effects of replacing soybean meal with cotton meal and supplementing benzoic acid on the relative abundance cluster analysis of PICRUSt2 functional annotation of pigeon intestinal flora. PICRUSt2 functional annotation clustering heat map from gene function (COG, KO) (Figures 5A,C), enzyme activity (EC) (Figure 5B), metabolic pathway (PWY) (Figure 5D) to protein domain (Pfam) (Figure 5E), gene family (TIGRFAM) (Figure 5F), there are a large number of inter-group difference units in all functional annotation types, indicating that the microbial communities in different groups are not only different in classification and composition, but also show obvious inter-group differentiation in metabolism, enzymology, molecular function and other multi-dimensional levels.

### 3.8 Effects of cotton protein and benzoic acid on LEfSe analysis of intestinal flora in pigeons

**FIGURE 6:**
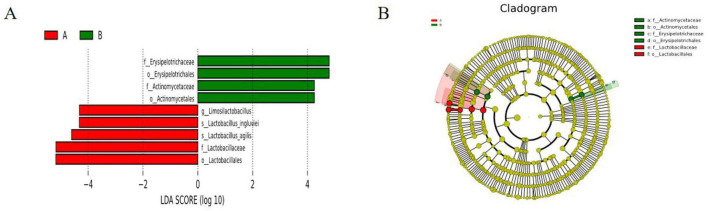
Effect of replacing soybean meal with cotton protein and supplementing benzoic acid on LEfSe analysis of intestinal flora in pigeons. **(A)** LDA (linear discriminant analysis) value distribution histogram. **(B)** Evolutionary branch diagram. A: Basic diet group; B: 5% cotton protein instead of soybean meal group; C: 5% cottonseed protein instead of soybean meal + 105 mg/kg benzoic acid group.

Figure 6 shows the effect of replacing soybean meal with cotton meal and supplementing benzoic acid on LEfSe analysis of pigeon intestinal flora. LDA (linear discriminant analysis) value distribution histogram (Figure 6A) analysis successfully identified the key biomarkers that distinguish the microbial communities of group A and group B. Group B was characterized by lactic acid bacteria and related species, while group A was significantly enriched in erysipelothrix and actinomycete. These markers not only explain the fundamental differences between the two groups of communities in terms of taxonomy, but also indicate potentially different metabolic functions and host interaction patterns.

Figure 6B shows the evolutionary branch diagram. The differences in microbial communities between groups A and B are reflected in multiple evolutionary independent groups: group A is characterized by Actinomycetes and Erysipelothrixes, and group B is centered on Lactobacillus. This differential distribution indicates that the two groups of communities may follow different ecological adaptation strategies and functional metabolic pathways.

### 3.9 Effects of cotton protein and benzoic acid on quality control (QC) and PCA analysis of pigeons

**FIGURE 7:**
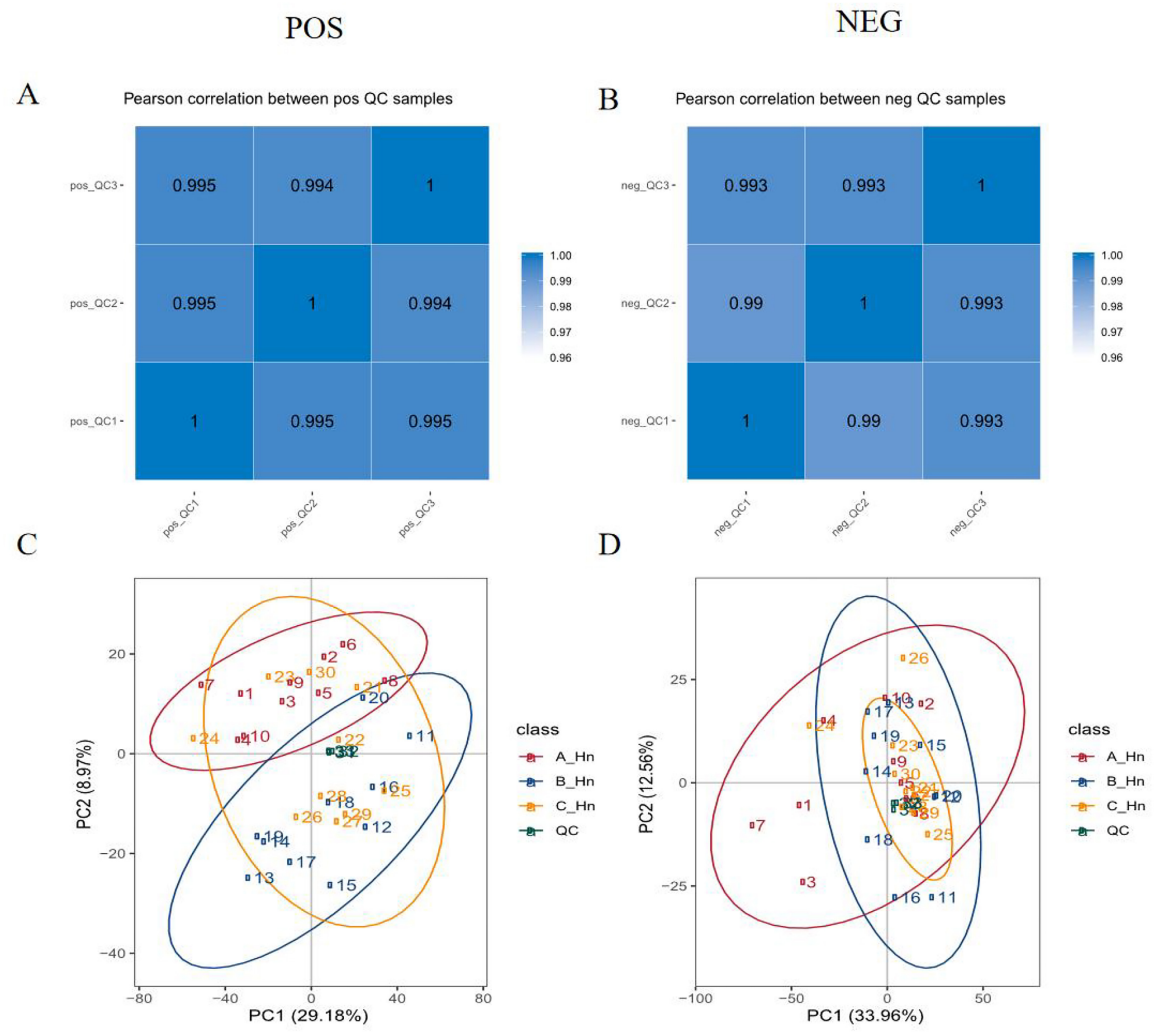
Effects of replacing soybean meal with cotton protein and supplementing benzoic acid on quality control (QC) and PCA analysis of pigeon samples. **(A,B)** Sample quality control analysis. **(C,D)** Total PCA analysis chart. A: Basic diet group; B: 5% cotton protein instead of soybean meal group; C: 5% cottonseed protein instead of soybean meal + 105 mg/kg benzoic acid group. The left side is the positive ion mode (POS), and the right side is the negative ion mode (NEG).

Figure 7 shows the effect of replacing soybean meal with cotton meal and supplementing benzoic acid on the quality control (QC) and PCA analysis of pigeon samples. We calculated the Pearson correlation coefficient between QC samples based on the relative quantitative values of metabolites. The higher the correlation of QC samples (|r| is closer to 1), the better the stability of the whole detection process and the higher the data quality (Figures 7A,B). According to the PCA model, the QC samples are closely clustered together, indicating that the stability and repeatability of this test are good. In the positive ion mode, the contribution rates of PC1 and PC2 were 29.18 and 8.97%, respectively (Figure 7C). In the negative ion mode, the contribution rates of PC1 and PC2 were 33.96 and 12.56%, respectively (Figure 7D). The separation between the two groups was obvious, indicating that the metabolites of the two groups were significantly different.

### 3.10 Effects of cotton protein and benzoic acid on partial least squares discriminant analysis (PLS-DA) in pigeons

**FIGURE 8:**
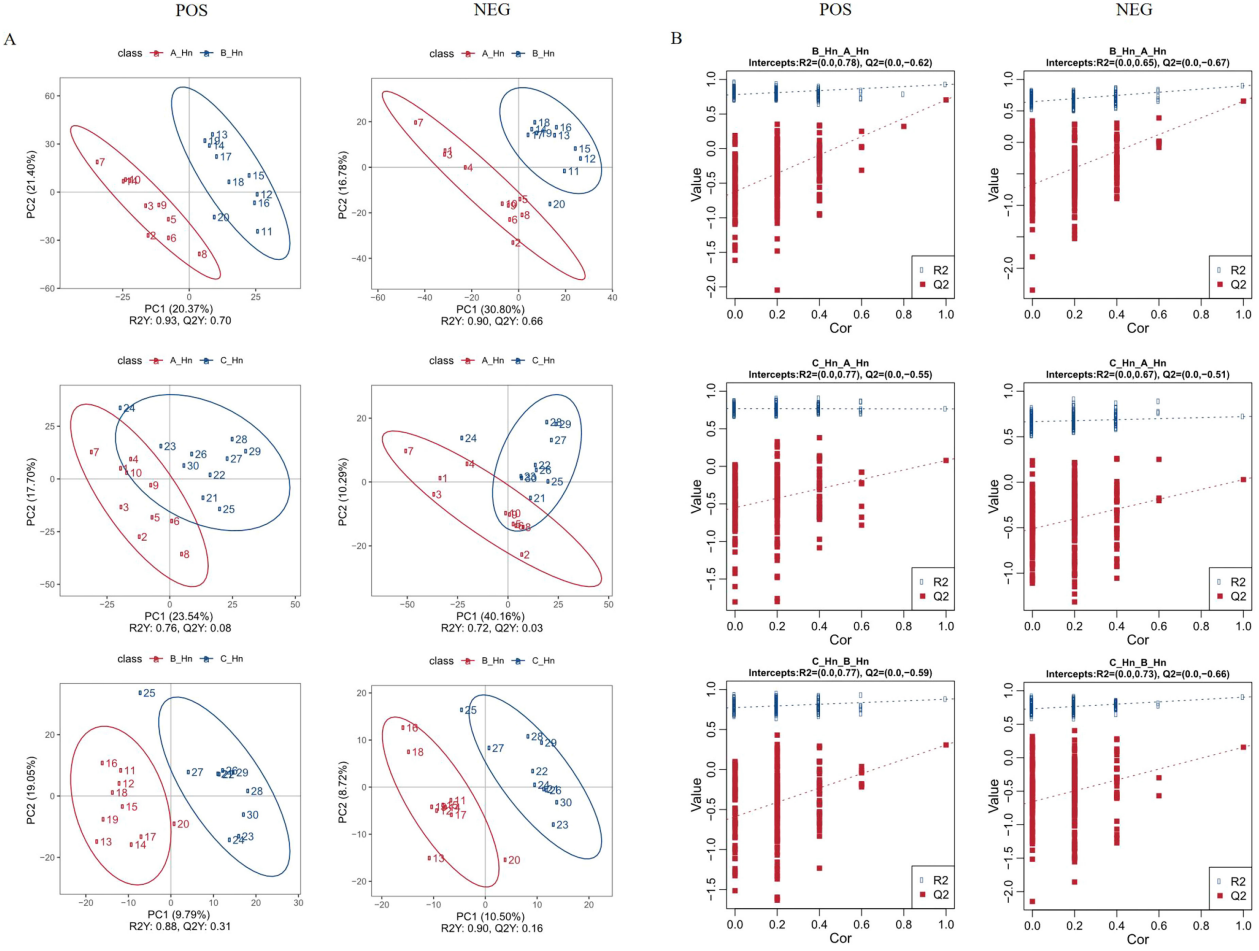
Effects of replacing soybean meal with cotton protein and supplementing benzoic acid on partial least squares discriminant analysis (PLS-DA) of squabs. **(A)** PLS-DA score scatter plot. **(B)** Sorting verification diagram. A: basic diet group; B: 5% cotton protein instead of soybean meal group; C: 5% cottonseed protein instead of soybean meal + 105 mg/kg benzoic acid group. The left side is the positive ion mode (POS), and the right side is the negative ion mode (NEG).

Figure 8 shows the effect of replacing soybean meal with cotton meal and supplementing benzoic acid on the partial least squares discriminant analysis (PLS-DA) of squabs. We applied supervised PLS-DA analysis to remove irrelevant difference information in PCA and further analyze the changes in the metabolic profiles of ileum contents in group A, group B, and group C. In this experiment, the R2Y of PLS-DA positive ion model was 0.93; 0.76; 0.88. Q2Y were 0.70; 0.08; 0.31. The R2Y of PLS-DA negative ion model was 0.90; 0.72; 0.90. Q2Y were 0.66; 0.03; 0.16. R2Y is > 0.5 and Q2Y is > 0.01, indicating that the model has high explanatory power and predictive ability. In the positive and negative ion mode, the separation of the two groups of data is better, which can effectively distinguish the samples of group A, group B, and group C (Figure 8A).

In order to further verify whether the PLS-DA model is over-fitting, 200 permutation tests were randomly performed. It can be seen from the permutation test model that the Q2 of the positive ion model is −0.62; −0.55; −0.59. The Q2 of the anion model is −0.67; −0.51; −0.66. The Q2 intercept is >0, which shows that the model does not have the phenomenon of “over-fitting,” indicating that the obtained data results are reliable and can be used for subsequent analysis of differential metabolite screening (Figure 8B).

### 3.11 Effects of cotton protein and benzoic acid on the overall distribution analysis of differential metabolites in pigeons

**FIGURE 9:**
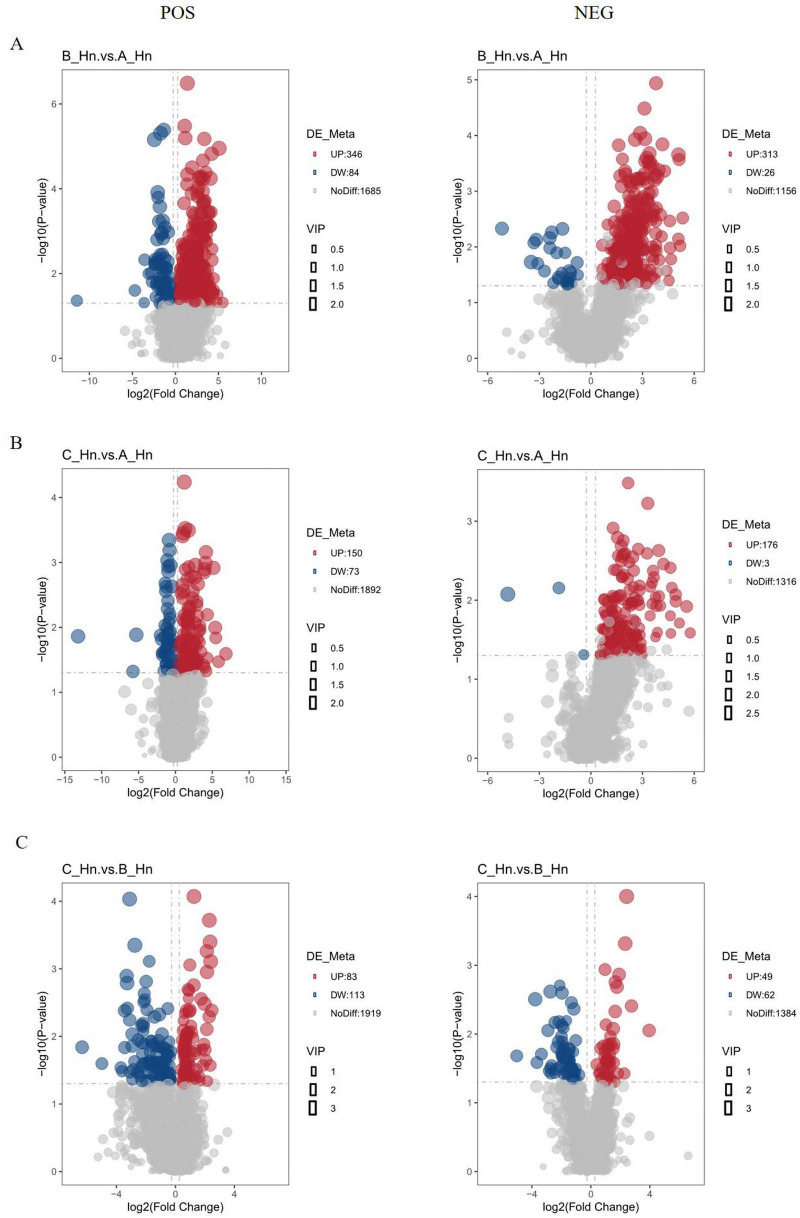
Effects of replacing soybean meal with cotton protein and supplementing benzoic acid on the overall distribution analysis of differential metabolites in pigeons. **(A)** Analysis of differential metabolites between group A and group B. **(B)** Analysis of differential metabolites between group A and group C. **(C)** Analysis of differential metabolites between group B and group C. A: basic diet group; B: 5% cotton protein instead of soybean meal group; C: 5% cottonseed protein instead of soybean meal+105 mg/kg benzoic acid group. The left side is the positive ion mode (POS), and the right side is the negative ion mode (NEG).

Figure 9 shows the results of the overall distribution analysis of differential metabolites in young pigeons by replacing soybean meal with cotton meal and supplementing benzoic acid. Non-targeted metabolites of ileum contents (LC-MS/MS) A total of 2,115 metabolites were detected in group A compared with group B in positive ion mode, and 430 differential metabolites were detected. Among them, 346 metabolites were up-regulated and 84 metabolites were down-regulated. There were 1,495 metabolites in the negative ion mode, and there were 339 differential metabolites, of which 313 metabolites were upregulated and 26 metabolites were down-regulated (Figure 9A). Compared with group A and group C, there were 2,115 metabolites in positive ion mode, and 223 differential metabolites, of which 150 metabolites were up-regulated and 73 metabolites were down-regulated. There were 1,495 metabolites in the negative ion mode, and 179 differential metabolites, of which 176 metabolites were up-regulated and 3 metabolites were down-regulated (Figure 9B). Compared with group B, there were 2,115 metabolites in positive ion mode and 196 differential metabolites in group C, of which 83 metabolites were up-regulated and 113 metabolites were down-regulated. There were 1,495 metabolites in the negative ion mode, and 111 differential metabolites, of which 49 metabolites were up-regulated and 62 metabolites were down-regulated (Figure 9C).

### 3.12 Effects of cotton protein and benzoic acid on KEGG enrichment analysis of differential metabolites in pigeons

**FIGURE 10:**
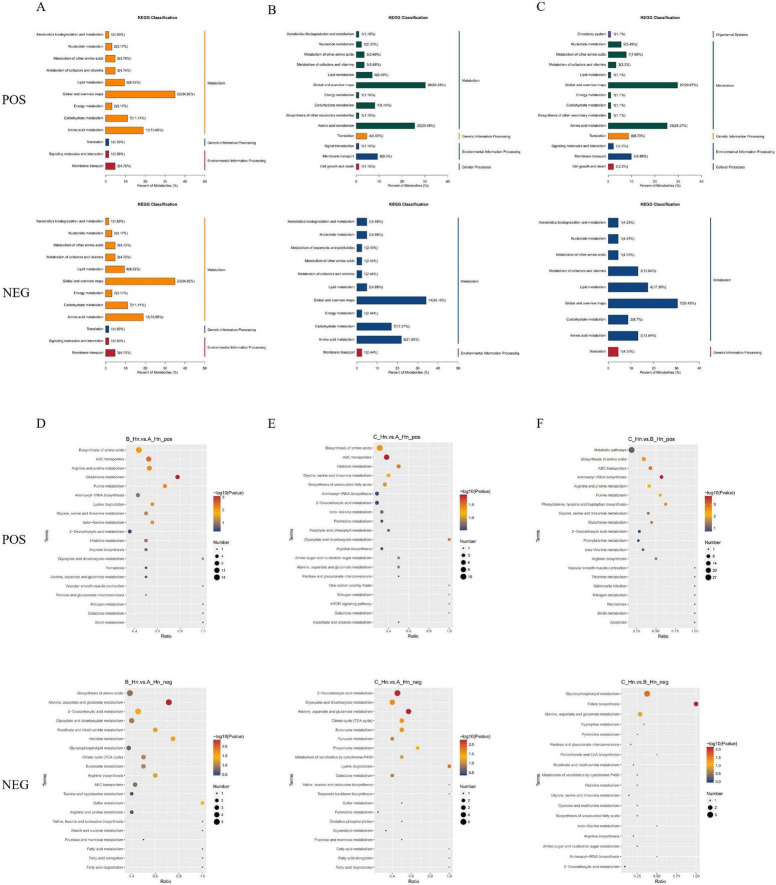
Effect of replacing soybean meal with cotton protein and supplementing benzoic acid on KEGG enrichment analysis of differential metabolites in pigeons. **(A–C)** KEGG classification of differential metabolites. **(D–F)** KEGG enrichment bubble diagram. Group A: basic diet group; B: 5% cotton protein instead of soybean meal group; C: 5% cottonseed protein instead of soybean meal+105 mg/kg benzoic acid group. The first line and the third line were positive ion mode (POS), the second line and the fourth line were negative ion mode (NEG).

Figure 10 shows the effect of replacing soybean meal with cotton meal and supplementing benzoic acid on KEGG enrichment analysis of differential metabolites in pigeons. Based on the metabolite classification data of KEGG database, we statistically analyzed the annotated differential metabolites (Figures 10A–C).

In order to further verify the function of KEGG enrichment of metabolic pathways, the analysis results showed that the differential metabolites were significantly enriched in 9 metabolic pathways. Among them, in the positive ion mode, it was significantly enriched in amino acid biosynthesis, ABC transporter, arginine and proline metabolism, histidine metabolic pathway (*P* < 0.05). In the negative ion mode, it was significantly enriched in alanine, aspartic acid and glutamic acid metabolism, 2-oxocarboxylic acid metabolism, glyoxylic acid and dicarboxylic acid metabolism, glycerophospholipid metabolism, and tryptophan metabolism pathways (*P* < 0.05) (Figures 10D–F).

### 3.13 Correlation analysis of microbiome and metabolome in young pigeons with cotton protein and benzoic acid

**FIGURE 11:**
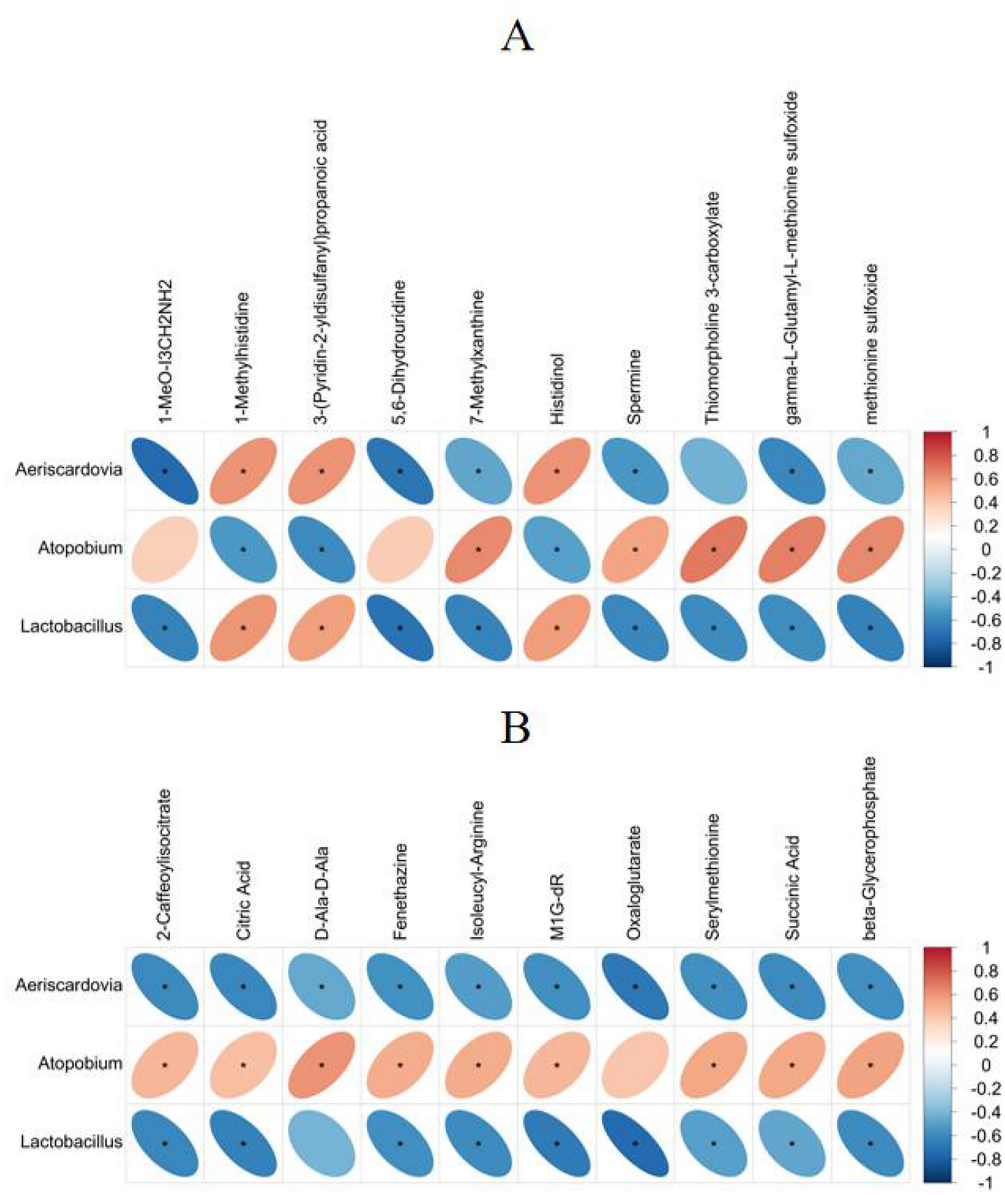
Results of combined microbial and metabolomic analysis of pigeon microbiota after cotton protein supplementation replacing soybean meal and benzoic acid supplementation. **(A)**: Positive ion mode. **(B)** Negative ion mode. The horizontal axis represents differential bacterial genera, the vertical axis represents differential metabolites, and the right legend indicates correlation coefficients, where red indicates positive correlation, blue indicates negative correlation, and *denotes statistical significance (*P* < 0.05).

We conducted correlation analysis between genera with significant differences at the genus level and metabolites with significant differences. The results are shown in the heatmap in Figure 11. The strongest correlations are as follows: Under the cation mode, Aeriscardovia exhibited significant positive correlations with 1-methylhistidine, 3-(pyridin-2-yl-disulfanyl) propanoic acid, and histidinol, while showing significant negative correlations with 1-MeO-I3CH2NH2, 5,6-dihydrouridine, 7-methylxanthine, spermine, gamma-L-glutamyl-L-methionine s-ulfoxide, and methionine sulfoxide (*P* < 0.05). Atopobium exhibited significant p-ositive correlations with 7-methylxanthine, spermine, thiomorpholine 3-carboxylate, gamma-L-glutamyl-L-methionine sulfoxide, and methionine sulfoxide, while s-howing significant negative correlations with 1-methylhistidine, 3-(pyridin-2-yl-disulfanyl) propanoic acid, and histidinol (*P* < 0.05). Lactobacillus demonstrated significant positive correlations with 1-methylhistidine, 3-(pyridin-2-yl-disulfanyl) propanoic acid, and histidinol, and negative correlations with 1-MeO-I3CH2NH2, 5,6-dihydrouridine, 7-methylxanthine, spermine, thiomorpholine 3-carboxylate, gamma-L-glutamyl-L-methionine sulfoxide, and methionine sulfoxide (*P* < 0.05).

In the anion mode, Aeriscardovia exhibited significant negative correlations with 2-Caffeoylisocitrate, Citric Acid, D-Ala-D-Ala, Fenethazine, Soleucyl-Arginine, M1G-dR, Oxaloglutarate, Serylmethionine, Succinic Acid, and beta-Glycerophosphate (*P* < 0.05). Atopobium showed significant positive correlations with 2-Caffeoylisocitrate, Citric Acid, D-Ala-D-Ala, Fenethazine, Soleucyl-Arginine, M1G-dR, Serylmethionine, Succinic Acid, and beta-Glycerophosphate (*P* < 0.05). Lactobacillus demonstrated significant negative correlations with 2-Caffeoylisocitrate, Citric Acid, Fenethazine, Soleucyl-Arginine, M1G-dR, Oxaloglutarate, Serylmethionine, Succinic Acid, and beta-Glycerophosphate (*P* < 0.05).

## 4 Discussion

### 4.1 Effects of cotton protein and benzoic acid on intestinal enzyme activities of pigeons

The digestion and absorption of most nutrients in poultry mainly occur in the small intestine, especially in the duodenum and the anterior jejunum. The absorption of nutrients in the small intestine depends on the decomposition of enzymes in the intestine. Therefore, the activity of intestinal digestive enzymes has become an important indicator to measure the digestion and absorption capacity of poultry (Xu et al., 2023; Li et al., 2024). At present, there are many reports on the effect of acidifier on intestinal enzyme activity (Sun et al., 2013). Sun et al. (2013) found that the activity of amylase and protease in the intestinal contents of 21-day-old broilers was significantly increased by feeding fermented cottonseed meal instead of soybean meal.

This study showed that there were significant differences in the activities of amylase and trypsin among different treatment groups, indicating that feed composition had a significant effect on the digestive function of young pigeons. From the perspective of amylase activity, the enzyme activity of group B and group C was significantly higher than that of group A, and that of group C was higher than that of group B, indicating that the substitution of cottonseed protein may help to improve starch digestion ability. This is largely consistent with previous research findings. And the addition of benzoic acid further enhanced this effect. The increase of amylase activity may mean the improvement of carbohydrate digestibility and be beneficial to energy utilization (Teng et al., 2024).

### 4.2 Effects of cotton protein and benzoic acid on intestinal length and intestinal pH of pigeons

The length of each segment in the intestine can directly reflect the development of the intestine and the ability of digestion and absorption of nutrients. The longer the length of the intestine, the longer the retention time of nutrients in the diet in the digestive tract, and the more nutrients are absorbed in the body, which will promote the growth and development of animals (Alyileili et al., 2020; Zhang H. et al., 2022). Intestinal pH is an important gastrointestinal factor for intestinal microorganisms, which is used to evaluate the absorption and utilization of nutrients in the digestive tract. Subtle changes outside the normal intestinal pH range can have a negative effect on the digestion and absorption of nutrients (Liu et al., 2022). Wang et al. (2015) found that the addition of organic acid mixture reduced the intestinal pH. Supplementation of antibiotics, probiotics and organic acids in the diet not only increased intestinal weight, but also reached the longest intestinal length on day 42.

The results of this study showed that the length of ileum in group A was significantly higher than that in group B and group C, while the length of duodenum and jejunum had no significant difference among the groups. Regarding intestinal pH levels, the pH values of all intestinal segments (duodenum, jejunum, ileum) in Groups B and C were significantly lower than those in Group A. This is not very similar to the findings of previous studies. This indicates that both cotton meal replacement of soybean meal and benzoic acid supplementation can reduce intestinal pH. The cotton meal replacement of soybean meal may affect intestinal structure and function by altering the composition of protein sources. Benzoic acid supplementation demonstrated significant intestinal acidification effects, which helps enhance digestive enzyme activity, inhibit pathogenic bacteria, and has a positive impact on the health and growth of young pigeons.

### 4.3 Effects of cotton protein and benzoic acid on intestinal histomorphology of pigeons

The small intestine is the main place for the digestion and absorption of animal nutrients. The morphological structure of the small intestine can directly reflect the strength of the digestion and absorption function. Small intestinal villi are the core part of the body to absorb and transport nutrients. Usually, the longer the intestinal villi, the higher the absorption efficiency of nutrients. The shorter the intestinal villi, the lower the absorption efficiency of nutrients (Li et al., 2024; Ismael et al., 2025). The crypt depth is an important indicator to reflect the maturation rate of intestinal epithelial cells. The deeper the crypt depth, the lower the maturation rate of intestinal epithelial cells (Bawish et al., 2023; Vieira et al., 2023). The ratio of villus height to crypt depth (VH/CD) in the small intestine can comprehensively reflect the digestion and absorption capacity of the small intestine. A higher ratio of villus height to crypt depth reflects higher nutrient absorption capacity and faster growth rate. On the contrary, it indicates that the digestion and absorption capacity is weakened (Hanim et al., 2023). Zhang Q. et al. (2022) found that coated sodium butyrate (CSB) treatment had a positive effect on intestinal morphology: superficial crypts were observed in the jejunum, and the villus height of ileum and jejunum was significantly increased. Hu et al. (2021) found that butyl butyrate three groups (TB3) and butyl butyrate four groups (TB4) mice at 21 and 42 days, the intestinal villus height and the ratio of villus length to crypt depth were significantly increased.

This study showed that the replacement of soybean meal with cottonseed protein was helpful to improve the structure of foregut, improve the efficiency of nutrient absorption and the stable state of immune function to a certain extent. This is largely consistent with previous research findings. Benzoic acid supplementation may indirectly promote the growth of villus by inhibiting harmful bacteria and reducing inflammatory response, thereby increasing the ratio of villus to crypt. It also improves intestinal health and absorptive capacity.

### 4.4 Effects of cotton protein and benzoic acid on the intestinal flora of pigeons

There are many complex, diverse and dynamic microbial communities in the intestinal tract of poultry, which play a vital role in the health and growth of animal bodies. In the process of nutrient metabolism and immunity, the intestinal microbial community can promote the digestion and absorption of nutrients in the body and stimulate the immune response of the host. Harmful microbiota in the gut has a direct or indirect negative effect on reducing digestibility, increasing cell renewal rate and the production of toxic metabolites (Wan et al., 2022; Deng et al., 2023). According to Wang et al. (2015), organic acids have no effect on the production performance of broilers, but reduce the number of intestinal microorganisms. Alpha diversity analysis showed that the higher the relative abundance of microbial community, Chaol index and ACE index, the higher the relative abundance of intestinal flora. The larger the Shannon diversity index and the smaller the Simpson index, the higher the diversity of intestinal microbial community. Mercy et al. (2025) Gwuegbu et al. found that the number of *E. coli* in broilers was significantly increased when the addition of benzoic acid reached 5.0 g/kg. In contrast, Salmonella and pathogenic bacteria were significantly reduced when the amount of benzoic acid added increased to 7.5 g/kg and 10 g/kg. The beneficial bacteria (lactic acid bacteria and bifidobacteria) peaked at 7.5 g/kg, indicating a positive change in microbial balance. The results of this study showed that there was no significant difference in Shannon index and Simpson index among the groups in the relative abundance of the ileum flora of the pigeons, suggesting that cotton meal replacement and supplement of benzoic acid can increase the relative abundance of the ileum microbial community of the pigeons.

Most of the studies on the flora in the intestinal contents are concentrated in the cecum, because the flora in the cecum is the most abundant, and the pigeon has the cecum at birth, but the cecum has been basically degraded when leaving the parent pigeon, and the functional cecum cannot be found in the pigeon, and the relative abundance of microorganisms in the back end of the intestine is relatively high, so this experiment collects the ileum contents of the pigeon to study the intestinal flora (Zhang et al., 2025). Studies by Nafady et al. (2025) have shown that dietary benzoic acid supplementation can significantly reduce the colonization of Salmonella in the intestinal tract of meat pigeons. Zhang et al. (2023) have found that dietary supplementation of benzoic acid plays an important role in regulating the microbial community of laying hens. At the phylum level, the results of Zhang et al. (2025) showed that among the hens in the benzoic acid, Enterococcus faecalis and essential oil complex group, compared with the basal diet group, Bacteroidetes, Firmicutes and Proteobacteria showed significant advantages at the phylum level. The specific performance is : the abundance of Bacteroidetes continues to rise, while Firmicutes and Proteobacteria show a downward trend. Bacteroidetes and Firmicutes are the key flora to maintain intestinal homeostasis, and their functions involve nutritional metabolism regulation and immune regulation. At the genus level, Liu et al. (2023) found that under the infection of coccidia and clostridium perfringens, feeding with benzoic acid and essential oil complex would lead to the transformation of intestinal microenvironment, which significantly increased the abundance of Bacillus and Lactobacillus.

In this experiment, the top 10 different phyla, classes, orders, families, genera and species of the ileum flora of the pigeons were identified. The results of this experiment showed that at the phylum level, all the content samples in the experimental group and the control group showed almost the same community structure. Firmicutes, Actinobacteria and Proteobacteria are the dominant flora, and the dominant flora can resist the colonization of foreign bacteria. The relative abundance of Firmicutes in the diet supplemented with 105 mg/kg benzoic acid was significantly higher than that in the control group, indicating that the supplementation of a certain concentration of benzoic acid in the diet can promote the reproduction of Firmicutes, which is conducive to the absorption and utilization of nutrients in the body, and the fermentation ability of benzoic acid can accelerate the biosynthesis and metabolism of amino acids.

### 4.5 Effects of cotton protein and benzoic acid on the metabolome of pigeons

Non-targeted metabolomics is a relatively new field of omics, which refers to the use of liquid chromatography-tandem mass spectrometry (LC-MS), gas chromatography mass spectrometry (GC-MS) and nuclear magnetic resonance (NMR). The dynamic changes of all small molecule metabolites (mainly endogenous small molecule compounds with a relative molecular weight of < 1,000 Da) before and after stimulation or disturbance in cells, tissues, organs or organisms were detected without bias. Differential metabolites were screened by bioinformatics analysis, and pathway analysis of differential metabolites was performed to reveal the physiological mechanism of their changes (Niu et al., 2019; Wu et al., 2025).

The main metabolites annotated in this experiment include L-γ-glutamyl-L-amino acid, glutathione, arginine, deoxyadenosine, 5-hydroxyisouric acid, quinolinic acid, carnosine, phosphoserine, choline, betaine, uric acid salt, citrulline, leucine, isonicotinic acid, bilirubin, and sphingosine. Crude protein is an import-ant part of animal diet. The difference of crude protein from different sources and nutrients in diet will have different effects on body metabolism. Hong et al. (2020) showed that, Tenebrio molitor larvae have good nutritional value, rich in protein (43.9∼51.0%) and fat (19.1∼36.7%), balanced amino acid ratio, and also contain functional substances such as antimicrobial peptides.

In this study, a total of 2,115 metabolites were identified in the ileum con-tents of pigeons by LC-MS technology, of which 430 were differential metabolites. Enrichment analysis of metabolic pathways showed that amino acid biosynthesis, ABC transporter, arginine and proline metabolism, histidine metabolism, alanine, aspartic acid and glutamic acid metabolism, 2-oxycarboxylic acid metabolism, glyoxylic acid and dicarboxylic acid metabolism, glycerophospholipid metabolism and tryptophan metabolism were significantly changed.

### 4.6 Effects of cotton protein and benzoic acid on the joint analysis of microbiome and metabolome in pigeons

We conducted correlation analyses between genera with significant differences at the genus level and metabolites with significant differences. The strongest correlations were as follows: Under the cation mode, Aeriscardovia showed significant positive correlations with 1-methylhistidine, 3-(pyridin-2-yl-disulfanyl) propanoic acid, and histidinol, while exhibiting significant negative correlations with 1-MeO-I3CH2NH2, 5,6-dihydrouridine, 7-methylxanthine, spermine, gamma-L-glutamyl-L-methionine sulfoxide, and methionine sulfoxide. Atopobium exhibited significant positive correlations with 7-methylxanthine, spermine, thiomorpholine 3-carboxylate, gamma-L-glutamyl-L-methionine sulfoxide, and methionine sulfoxide, while showing significant negative correlations with 1-methylhistidine, 3-(pyridin-2-yl-disulfanyl) propanoic acid, and histidinol. Lactobacillus demonstrated significant positive correlations with 1-methylhistidine, 3-(pyridin-2-yl-disulfanyl) propanoic acid, and histidinol, and negative correlations with 1-MeO-I3CH2NH2, 5,6-dihydrouridine, 7-methylxanthine, spermine, thiomorpholine 3-carboxylate, gamma-L-glutamyl-L-methionine sulfoxide, and methionine sulfoxide.

In the anion mode, Aeriscardovia exhibited significant negative correlations with 2-Caffeoylisocitrate, Citric Acid, D-Ala-D-Ala, Fenethazine, Soleucyl-Arginine, M1G-dR, Oxaloglutarate, Serylmethionine, Succinic Acid, and beta-Glycerophosphate. Atopobium showed significant positive correlations with 2-Caffeoylisocitrate, Citric Acid, D-Ala-D-Ala, Fenethazine, Soleucyl-Arginine, M1G-dR, Serylmethionine, Succinic Acid, and beta-Glycerophosphate. Lactobacillus demonstrated significant negative correlations with 2-Caffeoylisocitrate, Citric Acid, Fenethazine, Soleucyl-Arginine, M1G-dR, Oxaloglutarate, Serylmethionine, Succinic Acid, and beta-Glycerophosphate.

## 5 Conclusion

Under the experimental conditions, substituting soybean meal with cotton meal and supplementing with benzoic acid in juvenile pigeon diets significantly influenced intestinal tissue morphology, ileal microbiota, and metabolome. The changes in ileal bacteria following a 105 mg/kg benzoic acid supplementation showed strong correlations with metabolites in the ileum. Notably, the number of Lactobacillus at the taxonomic level affected histidine metabolism, indicating that benzoic acid supplementation primarily modulates amino acid metabolism and related metabolic pathways in the ileum of meat pigeons. This regulation of ileal microbiota structure promotes intestinal health and enhances the digestion and absorption of nutrients in juvenile pigeons.

## 6 Limitations

Several limitations of the present study should be acknowledged. First, the sample size was relatively limited, which may restrict the generalizability of our conclusions to broader populations or experimental conditions. Second, only selected indicators related to intestinal contents metabolism were detected in this work; more omics analyses such as transcriptomics and proteomics are needed to systematically clarify the underlying molecular mechanisms. Third, this study was conducted under controlled laboratory conditions, and *in vivo* physiological responses in complex practical environments remain to be further verified. Despite these shortcomings, our findings still provide valuable reference for subsequent relevant research.

## Data Availability

The original contributions presented in this study are included in this article/supplementary material, further inquiries can be directed to the corresponding author.
